# Modified Cap-Assisted Endoscopic Mucosal Resection Versus Endoscopic Submucosal Dissection for the Treatment of Rectal Neuroendocrine Tumors ≤10 mm: A Randomized Noninferiority Trial

**DOI:** 10.14309/ajg.0000000000001914

**Published:** 2022-08-23

**Authors:** Xuelian Gao, Shaohui Huang, Yusi Wang, Qun Peng, Weixin Li, Yingying Zou, Zelong Han, Jianqun Cai, Yuchen Luo, Yaping Ye, Aimin Li, Yang Bai, Ye Chen, Side Liu, Yue Li

**Affiliations:** 1 Department of Gastroenterology, Nanfang Hospital, Southern Medical University, Guangzhou, China;; 2 Graduate School of Biomedical Sciences, Icahn School of Medicine at Mount Sinai, New York, USA;; 3 Department of Pathology, Nanfang Hospital, Southern Medical University, Guangzhou, China; and; 4 Department of Gastroenterology, Shenzhen Hospital, Southern Medical University, Shenzhen, China.

## Abstract

**INTRODUCTION:**

Although recent guidelines recommend endoscopic resection of rectal neuroendocrine tumors (NET) ≤10 mm, there is no consensus on which endoscopic modality should be performed. We aimed to compare the safety and efficacy of modified cap-assisted endoscopic mucosal resection (mEMR-C) and endoscopic submucosal dissection (ESD) methods for the treatment of rectal NET ≤10 mm.

**METHODS:**

A randomized noninferiority trial comparing mEMR-C and ESD was conducted. The primary outcome was the histological complete resection rate; the secondary outcomes included *en bloc* resection rate, operation time, complications, and so on. Subgroup analyses and follow-up were also performed.

**RESULTS:**

Ninety patients were enrolled, and 79 patients with pathologically confirmed rectal NET were finally analyzed, including 38 cases of mEMR-C and 41 cases of ESD. Histological complete resection rate was 97.4% in the mEMR-C group and 92.7% in the ESD group. The noninferiority of mEMR-C compared with that of ESD was confirmed because the absolute difference was 4.7% (2-sided 90% confidence interval, −3.3% to 12.2%; *P* = 0.616). *En bloc* resection and successful removal of rectal NET were achieved in all patients. Advantages of mEMR-C over ESD included shorter operation time (8.89 ± 4.58 vs 24.8 ± 9.14 minutes, *P* < 0.05) and lower hospitalization cost ($2,233.76 ± $717.70 vs $2,987.27 ± $871.81, *P* < 0.05). Postoperative complications were recorded in 4 patients who received mEMR-C and 2 patients in the ESD group (11.5% vs 4.9%, *P* = 0.509), which were all well managed using endoscopy. Similar findings were observed when subgroup analysis was performed.

**DISCUSSION:**

mEMR-C is noninferior to ESD with a similar complete resection rate. In addition, mEMR-C had shorter procedure duration time and lower hospitalization costs.

**TRIAL REGISTRATION:**

ClinicalTrials.gov Identifier: NCT03982264.

## INTRODUCTION

Neuroendocrine tumors (NET) are heterogeneous neoplasms that arise from secretory cells of the diffuse neuroendocrine system and are characterized by a relatively indolent growth rate and the ability to secrete a variety of peptide hormones and biogenic hormones (1). The incidence and prevalence of NET are rising worldwide, and the rectum is one of the most common sites for gastroenteropancreatic NET (2).

Rectal NET usually appear as solitary, yellowish, and sessile polypoid lesions, most of which arise from the deep portion of glands and invade the muscularis mucosae and submucosa (1,3). According to the WHO 2019 classification of gastrointestinal tumors, NET are defined as malignant tumors (4), and for rectal NET, histological complete resection for those that could be resected is essential. The National Comprehensive Cancer Network consensus guideline indicates that tumors ≤10 mm in diameter can be resected using transanal or endoscopic excision (5). Furthermore, the European Neuroendocrine Tumor Society consensus guideline recommends endoscopic resection of NET measuring ≤10 mm without muscularis propria invasion or lymph node involvement (6). Many endoscopic methods for the management of small NET have been reported, including simple polypectomy, endoscopic mucosal resection (EMR), endoscopic submucosal resection with an endoscopic variceal ligation device, traditional cap-assisted EMR (EMR-C), and endoscopic submucosal dissection (ESD) (7–13). However, there is still no broad consensus on the specific endoscopic modality for small rectal NET (≤10 mm). Currently, EMR-C and ESD are the 2 modalities that are commonly applied in clinical practice, both of which demonstrate a satisfactory complete resection rate. In this study, we proposed a modified cap-assisted EMR method (mEMR-C) without submucosal injection.

In this study, we aimed to conduct a well-designed randomized controlled trial to evaluate the safety and efficacy of mEMR-C and ESD methods for the treatment of small rectal NET to provide high-quality evidence regarding optimal endoscopic management in this patient population.

## METHODS

### Study design

We conducted a single-center, prospective, randomized, noninferiority clinical trial to investigate whether mEMR-C was noninferior to ESD for the treatment of small rectal NET ≤10 mm at Nanfang Hospital of Southern Medical University. This study was approved by the Institutional Review Board of our institution and registered at clinicaltrials.gov (NCT03982264).

### Patients

Patients aged between 18 and 75 years, with a high suspicion or evidence of rectal NET measuring ≤10 mm assessed using EUS or CT, for whom both mEMR-C and ESD were deemed feasible by a multidisciplinary expert team, were recruited consecutively from September 2019 to January 2022. Exclusion criteria were lymph node metastases or distant metastases detected using endoscopic ultrasonography (EUS) or computed tomography (CT), inability to tolerate endoscopic resection, comorbidity of malignant tumor or other severe concomitant diseases, unsuitability for inclusion based on the multidisciplinary expert team team, and inability or unwillingness to provide written informed consent.

All patients signed a detailed informed consent form and were randomly assigned to the mEMR-C or ESD groups. The specific procedures of mEMR-C or ESD were performed by experienced endoscopists, who had performed at least 200 EMR cases and 100 ESD cases, and all the procedures were required to follow the standard procedures described further.

### Interventions

#### Procedure of mEMR-C.

**Figure 1.**
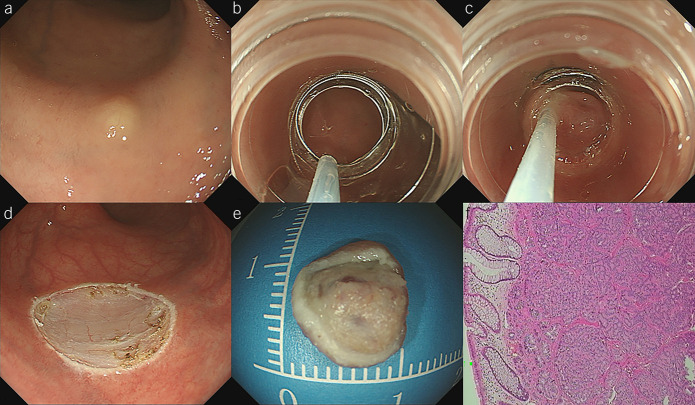
Modified cap-assisted endoscopic mucosal resection (mEMR-C). (**a**) This tumor appeared as a yellowish polypoid lesion whose surface was covered with normal mucosa. (**b**) The snare passed through the sheath and was looped along the inner groove of the cap. (**c**) The tumor was then suctioned into the cap, and the snare was tightened and closed under the base of the tumor. (**d**) After the tumor was removed, a clear resection surface can be observed, and the visible vessels were coagulated. (**e**) The resected specimen was retrieved and sent for pathological evaluation. (**f**) This tumor was pathologically confirmed rectal neuroendocrine tumor (NET) with negative resection margin.

A transparent cap with an inner groove (MH-593; Olympus, Tokyo, Japan) was attached to the forward-viewing colonoscope. After the endoscope was inserted into the rectum, a crescent-shaped electrosurgical snare was passed through the sheath and looped along the inner groove of the cap. No submucosal injection or lifting was performed with this technique to facilitate suction of the tumor into the cap and resection. The tumor was suctioned into the cap and grasped by tightening the snare. After confirming the appropriate snare placement, both the tumor and overlying mucosa were resected using electric cautery (Endocut Q, effect 2, VIO 200D; ERBE, Tübingen, Germany), and the resected tumor was sent for a pathological examination (Figure 1, see Video 1, Supplementary Digital Content 2, http://links.lww.com/AJG/C615). Endoscopic examination was repeated without a transparent cap to evaluate the wound carefully in cases of perforation or bleeding and to ensure the absence of residual tumor tissues. If there was spurting or active bleeding, hot forceps were used to stop the bleeding.

#### Procedure of ESD.

**Figure 2.**
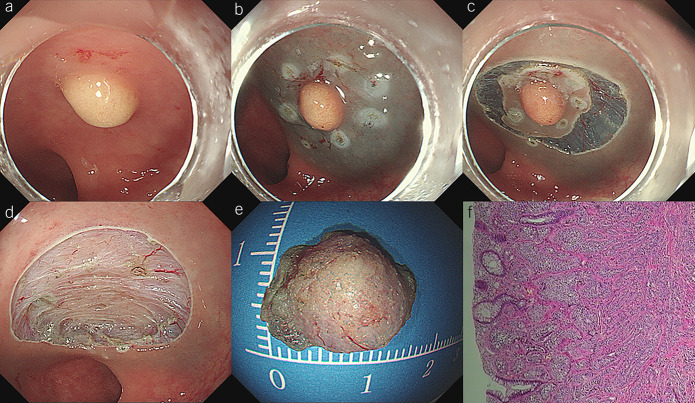
Endoscopic submucosal dissection (ESD). (**a**) This tumor appeared as a yellowish protruded polyp whose surface was covered with normal mucosa. (**b**) The periphery of the lesion was marked by coagulation, and diluted indicarminum saline was submucosally injected. (**c**) Mucosal incision and submucosal dissection were then performed. (**d**) After the tumor was removed, a clear resection surface can be observed. (**e**) The resection specimen was retrieved and evaluated. (**f**) This tumor was pathologically confirmed rectal neuroendocrine tumor (NET).

ESD was performed as the standard procedure and has been widely described and used. First, dots were marked approximately 5 mm from the periphery of the lesion using coagulation. Subsequently, a diluted sodium hyaluronate solution with indigo carmine dye was injected submucosally. Mucosal incision and submucosal dissection were performed using a T-Type ESD Knife (Micro-Tech, Nanjing, China) or Dual-knife (Olympus Medical, Tokyo, Japan). After resection was completed, all visible vessels on the artificial wound were thoroughly coagulated to prevent postoperative bleeding (Figure 2, Video 2, Supplementary Digital Content 3, https://webfiles.gi.org/videos\journals\ajg\AJG220499.mp4).

### Data collection

Data were collected by the investigators using standardized case record forms. Standard demographics of the patients were collected to assess the baseline comparability of the randomized groups, including sex, age, and tumor size, location, and depth predicted by preoperative EUS. Operation-related details, including operation time, cost, complications, *en bloc* resection, and pathologic complete resection rate, were also recorded. Other information included the pathological results, hospitalization costs, and duration of hospitalization.

### Outcomes and definitions

The efficacy was evaluated by assessing the rates of histological complete resection (R0), *en bloc* resection, and operation success, and the safety was evaluated by assessing the complications.

The primary endpoint was the histological complete resection (R0) rate. Histological complete resection was defined as complete single-piece (*en bloc*) resection of the targeted lesion with horizontal and vertical free margins. All resected tumors were evaluated by a single pathologist (Dr. Yaping Ye) who was blinded to the randomization and the specific resection technique.

The secondary endpoints included the following:

1. *En bloc* resection rate. *En bloc* resection was defined as complete single resection of the targeted lesion, irrespective of whether the basal and lateral tumor margins were infiltrated or undetermined.
2. Operation time, defined as the time required to complete the procedure, was taken from the installation of the snare in the mEMR-C or the first submucosal injection in ESD to the end of complete resection of the targeted area or a failure or complication of the procedure, which required discontinuation.
3. Success rate of operation, defined as the proportion of patients whose tumors were successfully resected in each group.
4. Complications, including intraoperative and postoperative complications, were defined as perforation or hemorrhage during or after the operation. Perforation was defined as an endoscopically visible hole in the rectal wall and/or postoperative clinical symptoms of peritonitis, perianal pain, and/or radiological evidence of free air under the diaphragm. Bleeding was classified as procedural or delayed. Procedural bleeding was defined as arterial bleeding or active oozing for more than 30 seconds during the operation, which required endoscopic, radiological, or surgical intervention. Delayed bleeding was defined as postoperative clinical symptoms, such as hematemesis and melena within 14 days after the procedure, which required endoscopic, radiologic, or surgical intervention.
5. Histopathologic grade included NET grade 1, NET grade 2, NET grade 3, and NEC, according to the 2019 WHO classification.
6. The length of hospitalization was defined as the total number of days from hospitalization to discharge.
7. Operation cost was defined as the cost of mEMR-C or ESD procedures, except the cost of other endoscopic procedures.
8. Total hospitalization cost was defined as the total cost of hospitalization.

### Follow-up

All patients underwent postoperative follow-up on days 3 and 14 after surgery to identify possible complications. As recommended by the National Comprehensive Cancer Network guidelines, no additional follow-up is required for patients with G1 NET with negative margins (5). However, those with positive margins were required to receive further endoscopic surveillance 6 months after surgery to reevaluate the residual or recurrent neoplasia, and if necessary, EUS and additional endoscopic resection were performed.

### Sample size calculation

The sample size was calculated to demonstrate the noninferiority of mEMR-C compared with ESD for the treatment of rectal NET for the complete resection rate. The null hypothesis was that the difference between the histological complete resection rates of the 2 groups would be 15% or more (noninferiority margin). This margin is based on a combination of clinical judgment and statistical reasoning. The reported histological complete resection rate of ESD in the literature is approximately 93.8% (12). A one-tailed sample size calculation was performed with a type I error rate (α) of 0.05 to obtain 80% power to show that the difference in histological complete resection rate was <15%, and the estimated sample size was 41 patients for each group, which was performed using PASS 15.0. With a 10% expected dropout rate, total recruitment was set at 45 patients for each group.

### Randomization

All enrolled participants were assigned to receive either mEMR-C or ESD in a ratio of 1:1 using simple randomization. The random allocation sequence was generated in advance, using a random number generator. We used sequentially numbered sealed opaque envelopes to conceal allocation. The relevant envelope was opened by the research fellow before the procedure for patients who met the criteria for study inclusion and had signed the informed consent. The treatment allocation was not masked to clinicians or patients for infeasibility.

### Statistical analysis

Continuous variables were summarized as the mean and SD values. Independent sample Student *t* tests were used to compare normally distributed and homoscedastic variables between the groups. Otherwise, we used the Mann-Whitney *U* test. Categorical variables were reported as the number of cases and percentages, and comparisons between groups were performed using the χ^2^ tests (or the Fisher exact test for smallest count <5). All *P* values provided were 2-sided, and *P* values < 0.05 for 2-tailed tests were considered significant. Noninferiority for the primary outcome was assessed by constructing a 2-sided 90% CI of the point difference between R0 resection rate in the mEMR-C group and in the ESD group. Noninferiority of mEMR-C would be considered if the lower bound of the CI for the difference of complete resection rate was greater than −15%. We also conducted subgroup analysis according to tumor sizes (tumor > 5 mm subgroup and ≤5 mm subgroup). All statistical analyses were performed using SPSS version 25.0 (IBM, Armonk, NY) and R4.1.1 (The R Foundation for Statistical Computing).

## RESULTS

### Patients' characteristics

**Figure 3.**
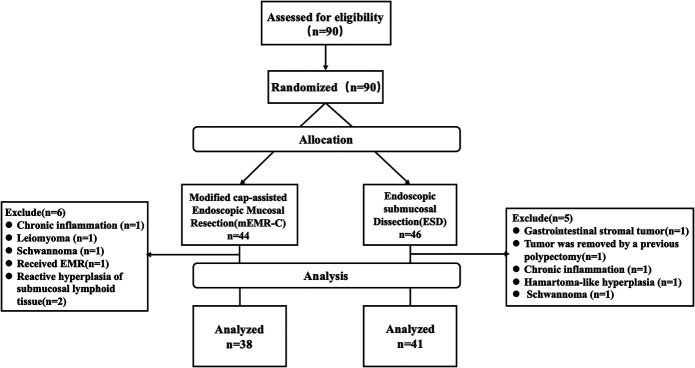
Consort flow diagram of patient enrollment.

Between September 2019 and January 2022, 90 patients with a high suspicion of small rectal NET were enrolled and randomly assigned to the mEMR-C group (44 patients) or the ESD group (46 patients). Finally, 79 patients with pathologically confirmed rectal NET were included in the analysis: 38 in the mEMR-C group and 41 in the ESD group (Figure 3).

As summarized in Table 1, the demographic and baseline characteristics of the participants were similar between the 2 groups.

**Table 1.**
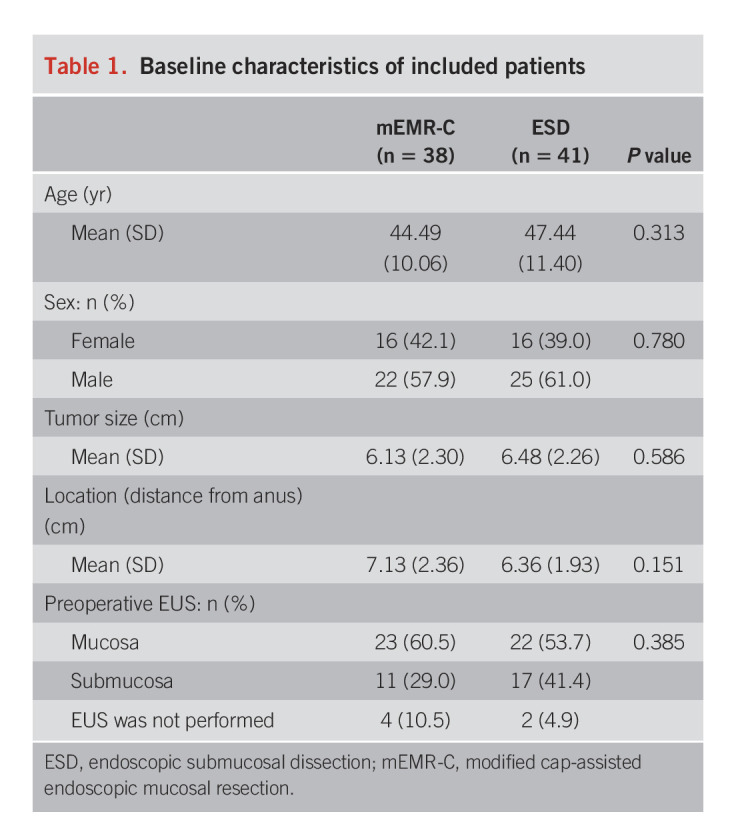
Baseline characteristics of included patients

### Intervention and hospitalization characteristics

**Figure 4.**
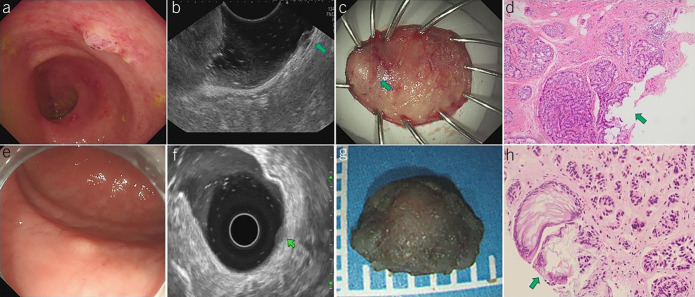
Positive resection margins were observed in modified cap-assisted endoscopic mucosal resection (mEMR-C) (**a**–**d**) and endoscopic submucosal dissection (ESD) (**e**–**h**) procedure. (**a**) The endoscopic view of the tumor. (**b**) Preoperative EUS indicated the tumor depth of submucosal layer. (**c**) The resected specimen was examined and the tumor (green arrow) located at the edge of the specimen. (**d**) This tumor was pathologically confirmed rectal neuroendocrine tumor (NET) with positive vertical margin (green arrow). (**e**) Endoscopic view of the tumor. (**f**) Preoperative EUS indicates tumor depth in the submucosal layer. (**g**) The resected specimen was examined, and the capsule of the tumor appeared incomplete. (**h**) The tumor was pathologically confirmed as a rectal NET with a positive vertical margin (green arrow).

The complete resection rate, which was the primary endpoint, was achieved in 37 of the 38 patients (37/38, 97.4%) in the mEMR-C group and in 38 of the 41 patients (38/41, 92.7%) in the ESD group. A positive vertical margin was observed in one patient in the mEMR-C group and in 3 patients in the ESD group (Figure 4). The difference in the complete resection rate between the study arms was 4.7%. The noninferiority of mEMR-C compared with ESD was shown because the 90% CI of the absolute difference was below the predefined margin (−3.3% to 12.2%, *P* = 0.616).

Regarding other procedural characteristics, successful resection and *en bloc* resection were achieved in all 79 patients. The mean operation time was significantly shorter in the mEMR-C group than in the ESD group (8.89 ± 4.58 vs 24.80 ± 9.14 minutes, *P <* 0.05). None of the 79 patients had intraoperative complications, including bleeding or perforation. Regarding postoperative complications, delayed bleeding was recorded in 3 patients (3/38, 7.89%) in the mEMR-C and in 2 patients (2/41, 4.88%) in the ESD groups, which were all successfully managed by subsequent endoscopy. Delayed perforation was observed in one patient in the mEMR-C group on the first postoperative day and was successfully closed using endoscopic clips.

The operation cost was significantly lower in the mEMR-C group than in the ESD group ($815.78 ± $167.50 vs $1,400.40 ± $424.93, *P* < 0.05). In addition, compared with the ESD group, the total hospitalization cost was significantly lower in the mEMR-C group ($2,233.76 ± $717.70 vs $2,987.27 ± $871.81, *P* < 0.05).

There were no significant differences between the 2 groups in the mean length of hospitalization (6.24 ± 2.78 days in the mEMR-C group vs 5.73 ± 2.23 days in the ESD group, *P* = 0.427). Pathological results were available for all patients. As summarized in Table 2, 89.5% (34/38) were G1 grade, and 10.5% (4/38) were G2 grade in the mEMR-C group, whereas 90.2% (37/41) were of G1 grade and 9.8% (4/41) were of G2 grade in the ESD group. The pathological results were similar between the 2 groups (*P* = 1.000), and most tumors were classified as G1 grade according to the staging system for NET of the American Joint Committee on Cancer.

**Table 2.**
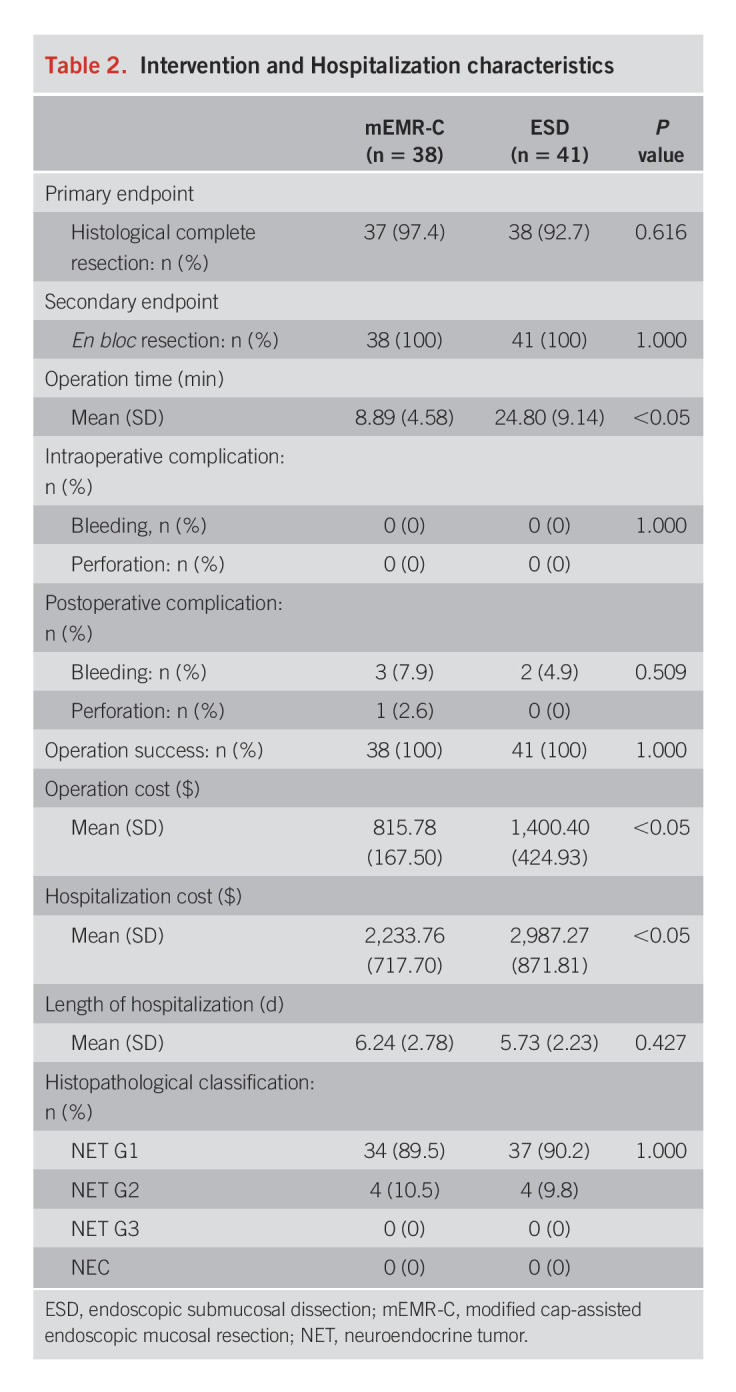
Intervention and Hospitalization characteristics

Follow-up colonoscopy was conducted for all patients with positive margins, and recurrence was not observed during the median follow-up period of 8.25 months in 4 patients (Table 3).

**Table 3.**
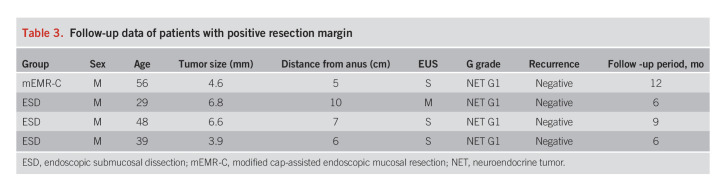
Follow-up data of patients with positive resection margin

### Subgroup analysis

Subgroup analyses according to tumor size were also conducted. No statistically significant differences were found in the demographic and baseline characteristics of the groups, either in the subgroup of patients with tumor size ≤5 mm or in the subgroup of patients with tumor size >5 mm. (see Supplementary Table 1, Supplementary Digital Content 1, http://links.lww.com/AJG/C614).

Histological complete resection rates were similar for tumor size ≤5 mm (mEMR-C, 93.3%; ESD, 94.1%, *P* = 1.000) and for tumor size >5 mm (mEMR-C, 100%; ESD, 91.7%, *P* = 0.489). The difference in complete resection rate was −0.8% (2-sided 90% CI, −14.9% to 13.4%) in the ≤5 mm group and 8.3% (2-sided 90% CI, −0.9% to 17.6%) in the group with a tumor size >5 mm. Consequently, mEMR-C was not inferior to ESD for the treatment of both rectal NET ≤5 mm or those >5 mm. In both subgroups, the mean operation time of mEMR-C was shorter, and the medical cost was lower (see Supplementary Table 2, Supplementary Digital Content 1, http://links.lww.com/AJG/C614).

## DISCUSSION

Small rectal NET (≤10 mm) could be removed endoscopically (5,6). To date, the standard technique for the endoscopic resection of small rectal NET remains unclear due to a lack of high-quality evidence. This study was the first randomized controlled trial to compare the safety and efficacy of mEMR-C with ESD for the treatment of small rectal NET. As shown by our data, mEMR-C was noninferior to ESD for the removal of rectal NET ≤10 mm, but mEMR-C was advantageous regarding shorter operation time and lower hospitalization cost.

Because most rectal NET invade the submucosa or deeper layers of the rectal wall (1,3), simple polypectomy and conventional EMR may not be suitable for incomplete histologic resection. Son et al. (14) showed that the pathological complete resection rate was 30.9% for conventional polypectomy. Yang et al. (12) reported that conventional EMR could not achieve a satisfactory histologic complete resection rate (76.8%) compared with EMR-C (94.1%) and ESD (93.8%). ESD, as a novel endoscopic method, was first developed in Japan to remove early gastric cancer and has gradually been confirmed to be safe and useful for rectal NET. Chen et al. (11) illustrated that ESD was effective for the resection of rectal carcinoids <20 mm and achieved a high R0 resection rate of 90.38%. Park et al. (10) demonstrated that EMR-C may be preferable for the treatment of small rectal NET ≤10 mm because it is technically easier and less time-consuming than ESD, with a higher histological complete resection rate (92.3%) than ESD (78.4%). However, most of these studies were retrospective with a limited number of cases, and some findings, such as the histological complete resection rate, were inconsistent.

In this study, we proposed a modified EMR-C without submucosal injection, which is much easier and faster than traditional EMR-C. We found that this technique was noninferior to ESD and could become one of the standard endoscopic techniques for the removal of small rectal NET. Our technique does not use injection or lifting because much more tissue together with the tumor can be aspirated into the cap, leading to adequate vertical resection depth. Another reason is that injection needle is not needed during this technique and medical cost can be decreased. In addition, the procedure is easier and faster to perform without using injection. Our data showed that the complete resection rates were 97.4% for mEMR-C and 92.7% for ESD. The reason why mEMR-C could achieve a high complete resection rate may be attributed to transparent cap aspiration, which ensured resection depth and free vertical margin. The transparent cap should be targeted at the tumor to ensure that the tumor can be completely aspirated into the cap. The incomplete resection case for the mEMR-C procedure in our study may be due to incorrect aspiration because the resected tumor was located at the edge of the specimen. Meanwhile, the reason for incomplete resection for ESD may be attributed to the tumor depth, and standard ESD cannot achieve a sufficient resection depth. In addition, mEMR-C was associated with a shorter operation time and lower hospitalization cost, although ESD is a new and advanced technique. The complication rates were low in both groups and were successfully managed using endoscopy.

This study represents the first randomized comparison between mEMR-C and ESD for the endoscopic treatment of small rectal NET with a good design and protocol. Our data confirmed that both mEMR-C and ESD are safe and effective methods, and mEMR-C was noninferior to ESD, which is currently widely used. The new and advanced ESD technique was not a better modality for small rectal NET, which may challenge the opinions of many experts in ESD.

Some limitations of this study should be considered when interpreting its findings. First, this was a single-center study conducted at a highly specialized tertiary referral center, and mEMR-C and ESD were performed by expert endoscopists, which may affect the generalizability of the findings. Second, we assigned all patients using computerized, simple randomization instead of stratified block randomization. Third, mEMR-C group had >10% expected dropout rate due to 5 cases of pathological non-NETs, which were finally excluded. In addition, this study compared mEMR-C only with ESD. Therefore, further studies comparing mEMR-C with other techniques are still required.

In conclusion, this study demonstrates that the complete resection rate of mEMR-C is high (97.4%) and is not inferior to that of ESD for the treatment of small rectal NET (≤10 mm). In addition, mEMR-C had shorter procedure duration time and lower hospitalization costs. A multicenter randomized controlled trial is needed to prove the universality and generality of these findings.

## CONFLICTS OF INTEREST

**Guarantor of the article:** Yue Li, MD.

**Specific author contributions:** Y.L. and X.G. conceived of the presented idea. S.H., Y.W., Q.P., Y.Z., and Y.C. performed the patient recruitment and data collection. X.G. recorded all the CRF and S.H. verified the CRF. Y.L., Z.H. and J.C. performed the endoscopic resection. W.L. and X.G. analyzed all the data. Y.Y. evaluated all the pathological specimen. A.L., Y.B., Y.C. and S.L. supervised the findings of this work. All authors discussed the results and contributed to the final manuscript.

**Financial support:** None to report.

**Potential competing interests:** None to report.

**Study Highlights**

**WHAT IS KNOWN**

✓ Recent guidelines recommend the resection of rectal neuroendocrine tumors (NET) measuring ≤10 mm without muscularis propria invasion or lymph node involvement.
✓ However, there is still no broad consensus on the specific endoscopic modality for the treatment of small rectal NET measuring ≤10 mm without muscularis propria invasion or lymph node involvement.

**WHAT IS NEW HERE**

✓ We proposed a modified cap-assisted endoscopic mucosal resection (mEMR-C) method and compared it with ESD for the treatment of small rectal NET.
✓ This randomized controlled trial confirmed that mEMR-C was noninferior to ESD in managing rectal NET measuring ≤10 mm, with a histological complete resection rate of 97.4%. In addition, mEMR-C has advantages over ESD, including a shorter operation time and lower hospitalization cost.
